# Factors Influencing Patient Adherence to Tuberculosis Treatment in Ethiopia: A Literature Review

**DOI:** 10.3390/ijerph17155626

**Published:** 2020-08-04

**Authors:** Zekariyas Sahile Nezenega, Lua Perimal-Lewis, Anthony John Maeder

**Affiliations:** 1 Department of Public Health, Ambo University, Ambo, Ethiopia; 2 Flinders Digital Health Research Centre, College of Nursing & Health Sciences, Flinders University, Adelaide SA 5001, Australia; anthony.maeder@flinders.edu.au; 3 Flinders Digital Health Research Centre, College of Science & Engineering, Flinders University, Adelaide SA 5001, Australia; lua.perimal-lewis@flinders.edu.au

**Keywords:** tuberculosis treatment, medication adherence, Ethiopia

## Abstract

Background: Tuberculosis (TB) is a major global public health problem and one of the leading causes of death among infectious diseases. Although TB can be cured with first-line antibiotics treatment of 6 months regimen, non-adherence to the treatment remains the main challenge for TB prevention and control. Interventions to promote adherence need to address multiple underlying factors linked to non-adherence, which requires a synthesis of studies to understand these factors in the local context. Our review accordingly examines these factors for TB treatment in Ethiopia. Methods: Articles were searched from PubMed and ScienceDirect databases, as well as manual searches through Google and Google Scholar search engines. Both quantitative and qualitative studies that showed factors associated with or reasons for non-adherence, default or loss to follow up from TB treatment were included. A total of 276 articles were screened, and 29 articles were ultimately included in the review. Findings: The extracted factors were synthesized thematically into seven dimensions of patient-centred, social, economic, health system, therapy, lifestyle, and geographic access factors. More than 20 distinct factors were identified under these headings. Some of these factors may also apply quite widely in other settings, with greater or lesser influence, but some are particularly applicable to the Ethiopian setting. Conclusion: Helping patients to achieve full adherence to TB medication is a complex problem as it is influenced by interplay between many factors. Healthcare managers, providers, and researchers need to consider and address multiple underlying factors when designing adherence interventions. This work provides a reference set of such factors for Ethiopian interventions.

## 1. Introduction

Tuberculosis (TB) is an infectious disease that is one of the major causes of death, being in the top ten causes of death worldwide and the leading cause of death from infectious disease, ranking above HIV/AIDS in 2018. Globally, one-fourth of the population is either infected with TB or at risk of developing the disease, with an estimated 10 million people infected with TB worldwide in 2018 [1]. Ethiopia has achieved a 50% reduction of TB through the Millennium Development Goals (MDGs) [2]. However, Ethiopia remains one of 14 high-burden TB countries for TB, TB-HIV, and Multi-drug-resistant tuberculosis (MDR-TB). An estimated 165,000 cases of TB incidence were reported for Ethiopia in 2018 [1].

Although TB can be cured with first-line antibiotics treatment of 6 months regimen, non-adherence is the main challenge for TB control and prevention programs. The patient needs to take >90% of TB medication to facilitate TB cure, and a patient who takes at least 95% is said to be ‘high adherence’. Treatment default is defined by the World Health Organization as a patient who interrupts treatment for 2 or more months [3]. The default rate is thus a crude method to monitor adherence [4]. Non-adherence to TB treatment increases the risk of morbidity, mortality, and drug resistance at both the individual and community level [5].

The World Health Organization has recommended Direct Observation of Treatment (DOT) by a trained supervisor, in which a healthcare worker watches the patient take the medication every day, to ensure adherence to treatment [6]. However, implementing DOT in Ethiopia is challenging for both the patient and healthcare provider. For example, one study conducted in Addis Ababa found patients reported that a daily visit to a health facility for the first two months was very difficult for a range of reasons, including severe illness at the initiation of treatment, distance too far for walking, and high transportation cost. Because of these challenges, DOT has not been implemented on a daily basis in Ethiopian standard care after the first two months of treatment [7].

A systematic review found that the pooled prevalence of non-adherence to TB treatment in Ethiopia was 21.3%. Forgetfulness, fear of drug side-effect, waiting time for 1 h or more during the service, and feeling a long distance to health facility were identified as factors associated with this non-adherence [8]. Another systematic review in the same setting found that the pooled prevalence of non-adherence to TB treatment and loss to follow-up were 20% and 5%, respectively. Being TB-HIV-co-infected, transport costs, lack of knowledge, drug side-effect, educational status, forgetfulness, being in continuation phase, perceived physical and psychological barriers, and psychological distress were identified as associated factors [9].

Interventions to promote adherence require addressing multiple components to overcome the barriers to adherence [10,11]. This requires a synthesis of studies to understand the causal factors for non-adherence to TB treatment in the local context. The above-mentioned systematic reviews and meta-analysis conducted in Ethiopia by Zegeye et al. (2019) [8] and Tolla et al. (2019) [9] had their main objective to estimate the pooled prevalence of non-adherence to TB treatment. The associated factors for non-adherence to TB treatment were not comprehensively identified by them, and their reviews included only quantitative studies.

This literature review synthesises both qualitative and quantitative studies to thematically present multiple factors that have been identified as influencing non-adherence to TB treatment in Ethiopia. This includes all types of TB such as active TB, latent TB, and TB-HIV co-infected patients. Therefore, this review could help healthcare managers, providers, and researchers to design and implement adherence interventions based on established contextual factors rather than ad hoc generalisations.

## 2. Methods

Research articles were searched for from PubMed and ScienceDirect databases, as well as manual search through Google and Google Scholar search engines. Search expressions were developed for TB medication adherence or loss to follow up or default from TB treatment that were published in the English language with no publication date restriction (see Table 1). The same expression of search strategy was used for all databases and search engines. Article searching was undertaken from 15 April to 5 May 2020.

**Figure 1:**
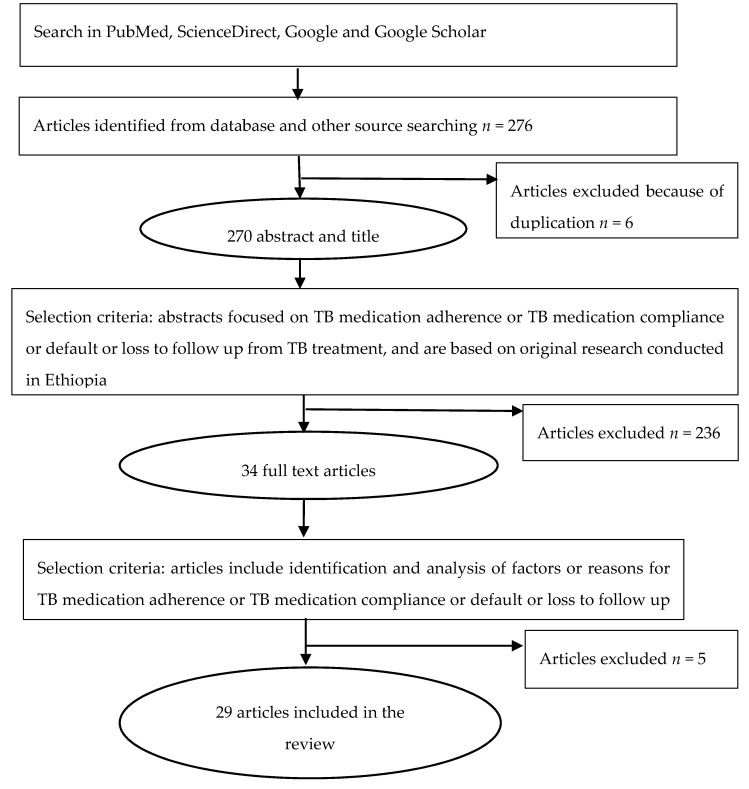
Flow chart for selection of reviewed articles.

Both qualitative and quantitative research articles were included, and articles that did not report original research were excluded. Articles that did not assess factors or reasons associated with TB medication non-adherence or default or loss to follow up from TB treatment were excluded, following the protocol of Figure 1. Data were extracted using an Excel template comprising the author, year, region, sample size, study design, population, and major findings. The article selection and data extraction were performed by the first author of this paper, and consistency was checked by the two other authors. Factors associated with TB treatment non-adherence were extracted from the quantitative type of studies, and reasons for non-adherence or default or loss to follow up were extracted from the qualitative type of studies. The extracted data were synthesized into groups based on the seven thematic dimensions of TB medication adherence factors proposed by Ogundele et al. (2015) [12]. These seven thematic dimensions are patient-centred, social, economic, health system, therapy, lifestyle, and geographic access factors.

## 3. Findings

A total of 276 research articles were screened, and 29 studies were ultimately included in this literature review. Of these, 6 articles were qualitative studies, 22 articles were quantitative studies (18 cross-sectional, 3 prospective cohort, and 1 case-control), and 1 article used a mixed-method design. These studies were conducted in Addis Ababa (9); Southern Nations, Nationalities, and Peoples’ Region (SNNPR) (7); Amhara (5), Oromia (5); and Tigray (3) regions of Ethiopia. Five studies were conducted among latent TB-HIV co-infected patients, and the remaining 24 studies were conducted among active TB infected patients. Approximately 7382 participants in total were involved in the studies reported in these 29 articles.

The synthesised findings from both quantitative and qualitative studies are presented below, grouped in seven dimensions of adherence influencing factors. The individual studies that showed factors or reasons linked with TB medication non-adherence are presented in Appendix A Table A1.

### 3.1. Patient-Centred Factors

Forgetfulness [13,14,15,16,17,18,19,20,21] and inadequate knowledge about tuberculosis and its treatment regimen [14,18,22,23,24,25] were the two major patient-centred factors. Three studies conducted in Oromia [26,27] and SNNPR [21] regions showed that the patient’s educational status was associated with non-adherence to TB medications: the more the patient was educated, the less likely was non-adherence to TB medication. Psychological distress was another factor: two studies conducted in Addis Ababa reported that this indirectly positively influences non-adherence to TB medication [28,29]. Another qualitative study conducted in Addis Ababa also stated that poor mental health status of a patient would make them reluctant to regularly attend follow up and clinic appointments [18].

### 3.2. Social Factors

Several studies reported that patients not getting social support from families and neighbours in remembering to take their medication, food, and financial assistance were the major social factors that influenced non-adherence to TB medication [18,19,24,26,30,31,32,33]. Additionally, one study in Addis Ababa conducted among latent TB-HIV co-infected patients reported that the patients’ friends’ decision to take the medication would make them less likely to be non-adherent to isoniazid preventive therapy (IPT) [34]. Another study among the same subjects and setting found that patients who were comfortable to take IPT in front of other people were less likely to be non-adherent [35]. Being busy with work [14] and away from home for work or other social-related activities were also found to influence non-adherence to TB medication [14,15,16]. Perceived and experienced stigma and discrimination also led the patient to non-adherence [18,36,37,38]. These particular factors were highly noted in studies conducted among TB-HIV co-infected patients [18,31,37,38]. As one study indicated, because of fear of stigma and discrimination, the patients were not disclosing their HIV status to their family, which in turn influenced their non-adherence to TB medication [18].

Beliefs about the disease and treatment, such as perceived wellness or cure, perceived risk, and perceived barriers over the benefits, were influencing factors for non-adherence to TB medication [13,26,28,29]. One study conducted in Addis Ababa reported that a patient’s belief in curability and severity of TB in the presence of HIV infection would make them less likely to be non-adherent [31]. Another study in Addis Ababa found that the perceived risk of discontinuing TB medication was the reason for adherence, while perceived wellness was the reason for patients have intention to discontinue TB treatment [13]. One study conducted in SNNPR also reported that belief in traditional healing influenced non-adherence to TB medication [36].

### 3.3. Economic Factors

The patient’s economic constraints (which impact the financial burden) was the main economic factor that influences non-adherence to TB medication [17,29,30,31]. Economic constraints limit the patient’s ability to have adequate food which influences non-adherence [31,36]. The cost of medication other than TB medications is also a factor for non-adherence in one study conducted in SNNPR. Another study conducted in SNNPR reported that the patient being not employed was associated with non-adherence [39].

### 3.4. Health System Factors

Poor healthcare provider–patient relationship with communication gaps was a major factor that influenced non-adherence to TB medication [14,31,32,36,39]. For example, one study conducted in Addis Ababa among loss-to-follow-up patients reported that the healthcare providers were seen as disrespectful of their patients and less committed to their profession [32]. The quality of healthcare service and a patient’s satisfaction with healthcare service affect non-adherence to TB medication [16,30,39]. When patients perceived that they received less professional care and less time spent with the healthcare providers, and waited a long time to get healthcare service, they were more likely to be non-adherent [16,32,39,40]. Health information/education is also crucial for adherence: a few studies showed that the patients who did not receive health information/education from health facilities were more likely to be non-adherent [15,18,22]. Additionally, one study done in Addis Ababa found that cues to action were reported as a factor for non-adherence [28]. Lack of supervision and healthcare providers incapable of managing the patient’s illness were also reported as influencing factors for interruption and default from TB treatment [38].

### 3.5. Therapy Factors

Many studies reported that drug side-effects were the major therapy-related reason for non-adherence to TB medication [15,16,23,24,26]. Pill burden was also reported as a factor for non-adherence to TB medication among active TB and TB-HIV co-infected patients [31,36]. The presence of more than one co-morbidity including TB-HIV co-infection was also reported as a factor for non-adherence to TB medication [14,16,20,26,37]. One study conducted in Addis Ababa also found that being on Antiretroviral Therapy (ART) was a factor for non-adherence to TB medication [29]. Symptom presence after initiation of anti-TB treatment and slow progression of the health status were also found as non-adherence factors [19,20,26,33]. Being in the continuation phase of the treatment (after the initial 2-month clinic-based treatment period) was a factor for non-adherence and default [14,20,24,34,41]. This might be due to the patient’s perceived wellness or cure because a daily DOT was not implemented after the first two months of treatment in Ethiopia.

### 3.6. Lifestyle Factors

Alcohol consumption was reported as a factor that influenced non-adherence to TB medication in several studies [14,16,27,29]. Cigarette smoking and khat (herbal stimulant) chewing were also found as factors associated with non-adherence [16,27].

### 3.7. Geographical Access Factors

Healthcare inaccessibility from residence location was a major geographical access factor for non-adherence and default from TB treatment [17,19,22,26,30,36,40]. Due to inaccessibility, patients were unable to keep regular clinic appointments and follow up treatment in two studies, conducted in Addis Ababa and Amhara regions [16,35]. Distance of the health facility was related to transportation cost, which was also a factor for non-adherence [21,22,25,33].

## 4. Discussion

Adherence to TB medication is a complex and dynamic matter as it is affected by multiple factors. This review has identified the range of multiple factors that have been found to affect non-adherence to TB medications in Ethiopia. The influence of these factors individually and in combination might vary from one social or geographic setting to the other. Healthcare managers need to consider the underlying factors for non-adherence to TB medication in the local setting (ideally using locally available evidence) when they design and implement an intervention.

Our literature review has found some similar influencing factors of non-adherence to TB treatment as those described in the previous two systematic reviews conducted in Ethiopia [8,9]. These factors were forgetfulness, inadequate knowledge about TB and its treatment regimen, psychological distress (poor mental health condition), perceived barriers, long waiting time, drug side-effects, TB-HIV co-infection, being on the continuation phase of treatment, healthcare inaccessibility, and travelling costs. Adherence interventions such as providing health information about TB and its treatment regimen, dealing with side-effects, providing reminders, and other health system interventions could be used to resolve these factors.

However, in this review, we have found several additional factors of non-adherence to TB treatment that were not identified by the previous two systematic reviews. These factors were lack of social support, being busy with work, being away from home, perceived and experienced stigma and discrimination, beliefs such as perceived wellness/cured, perceived risk, financial constraints to buy food and medication cost other than anti-TB, poor healthcare provider–patient relationship such as communication gaps, disrespecting patients, quality of healthcare service, patient satisfaction, lack of health information/education, pill burden, the persistence of symptoms after initiation of treatment, and use of substances such as alcohol, smoking, and khat chewing. These factors and the possible interventions to overcome the factors are discussed below.

We have found that lack of social support can influence non-adherence to TB medication. Thus, providing social support such as financial assistance for transport costs, food assistance, and reminders for medication intake from families and neighbours may help the patient to adhere to TB medication. In the same way, not getting social acceptance to take medication from families, friends, and neighbours can lead to non-adherence. A systematic review in developing countries reported the same finding [42]. Similarly, the perceived stigma and discrimination were reported as factors for non-adherence to TB medication. These were higher especially among TB-HIV co-infected patients. The other new social factors were being busy with work and away from home for work and social-related activities. These factors influence non-adherence might be due to the patient forgetting to take their medication when they are busy and away from home. A reminder from family or through mobile SMS text could be considered as a solution for these factors. Beliefs related to TB and its treatment can also influence non-adherence. This might be due to the patient’s perceived wellness or cure after taking some medications and thus interrupting their treatment. On the other hand, when the patient perceives that the disease is severe and the risk of discontinuing TB medication leads to poor health outcomes, their non-adherence to TB medication would be less likely. A systematic review conducted in developing countries also found that feeling well after the initiation of treatment was a factor for non-adherence to TB medication [42]. Providing patients with health information to change such wrong beliefs about the disease and its treatment could be considered as an adherence intervention to address these factors.

The financial constraints to get adequate food, transport cost, and medication cost other than anti-TB medication were found to influence non-adherence or loss to follow up from TB treatment. A systematic review conducted in developing countries also reported that the financial burden can cause TB medication non-adherence [42]. Financial assistance or support of food and transport and making all medications freely available to the patient may overcome this factor.

We also found that healthcare-system-related factors were other major factors for non-adherence to TB medication. Poor healthcare-provider relationship with communication gaps, disrespect of the patient, and lack of professional commitment influenced non-adherence to TB medication. A systematic review conducted in developing countries also found that poor patient–healthcare worker communication was a factor for non-adherence to TB medication [42]. The quality of health service as perceived by the patient and the patient satisfaction also influenced non-adherence to TB medication. It was also found that patients who did not receive health information/education were more likely to be non-adherent to TB medication. Therefore, health system interventions such as training for healthcare provider–patient communication and their relationships and health system strengthening to shorten waiting times and to raise the quality of health services could address these factors.

Pill burden was also a factor for non-adherence to TB medication both in TB and TB-HIV co-infected patients. TB-HIV co-infection has additional burden from the pills and drug side effects. Persistence of symptomatic conditions after initiation of TB treatment was another influence for non-adherence. This might be due to the patient’s belief in the curability of the disease becoming less when symptoms persist despite the treatment initiation. Health information about TB, its treatment, and side effects could reduce the influence of these factors. New drug investigations to reduce the drug side effect and to make a shorter treatment regimen could also be considered if it is possible.

Substance use such as alcohol use, cigarette smoking, and khat chewing was a factor for non-adherence to TB medication as reported by a few studies. The use of these substances might make the patient reluctant to follow the regular clinic appointment and follow up treatment. Other systematic reviews conducted in developing countries also showed that alcohol and tobacco were factors for non-adherence to TB medication [42]. Health information on how the use of substances affected treatment adherence and treatment outcome may help to resolve this factor.

## 5. Limitations

This review included studies conducted in Ethiopia; therefore the identified factors may not be generalizable to other settings. Adherence to TB medication is a complex problem as it is influenced by multiple factors so a single factor may not be shown as a cause–effect relationship.

## 6. Conclusions

This review describes more than 20 factors that influence adherence to TB treatment in Ethiopia, demonstrating that it is a complex problem that is affected by the interplay of multiple factors. We have found major additional factors for TB medication non-adherence or default or loss to follow up. These were social support from families and neighbours such as food support, reminders, and encouragement; being busy with work; being away from home; perceived and experienced stigma and discrimination; beliefs such as perceived wellness/cure; perceived risk; economic constraints for having adequate food and medication cost other than anti-TB medication; poor healthcare provider-patient relationships such as communication gaps, disrespecting patients, quality healthcare service, and patient satisfaction; health information/education, pill burden, the persistence of symptoms after treatment initiation; and use of substances. Healthcare managers, providers, and researchers need to address these underlying factors when they design and implement adherence interventions.

## Figures and Tables

**Table 1:** Searching strategy.

AND	(TB) OR (Tuberculosis)
AND	(“medication adherence”) OR (adherence) OR (“treatment adherence”) OR (“medication compliance”) OR (compliance) OR (default) OR (“loss to follow up”)
AND	Ethiopia

## Definition of Term

Adherence to a medication regimen is defined by Osterberg and Blaschke (2005) as “the extent to which patients take medications as prescribed by their health care providers”(10).

## Appendix A

**Table A1:** Summary of reviewed articles showing factors or reasons linked with TB medication adherence or default or loss to follow up from TB treatment.

First Author (Year)	Region	Study Design	Sample Size	Population	Associated Factors of Non-Adherence to TB Medication and Loss to Follow up/Default from TB Treatment
Daba et al. (2019) [27]	Oromia	Prospective cohort	268	Active TB patients	Khat (herbal stimulant) and alcohol use was associated with non-adherence with TB medication. Educational and occupational status were also associated with non-adherence to TB medication.
Sahile et al. (2018) [13]	Addis Ababa	A qualitative study	10	Active TB patients	Forgetfulness is the factor of TB medication non-adherence. The perceived risk and perceived wellness were also associated with TB medication non-adherence.
Mekonnen et al. (2018) [14]	Amhara	Cross-sectional study	314	Active TB patients	Being in the continuation phase of treatment, the presence of more than one co-morbidity, inadequate knowledge of TB and its treatment, poor patient–provider relationships, and alcohol use were statistically associated non-adherence to TB treatment. Additionally, forgetting to take medication, being busy with work, and out of home/town were the reasons mentioned by patients for non-adherence.
Gube et al. (2018) [40]	SNNPR	Cross-sectional study	271	Active TB patients	Drug side effects, a far distance from the health facility, and prolonged wait time to get healthcare service were statistically associated with non-adherence.
Woimo et al. (2017) [22]	SNNPR	Cross-sectional study	261	Active pulmonary TB patients	Inadequate knowledge of TB and its treatment, health information at each medication refill visits, a far distance from a health facility (more than 10 km), transportation cost, and cost of medications other than anti-TB.
Tola et al. (2017) [28]	Addis Ababa	Cross-sectional study	698	Active TB patients	Perceived barrier over and perceived benefits directly associated with non-adherence TB medication. Cue to action and psychological distress were indirectly influence non-adherence mediated through perceived barriers and benefits.
Tola et al. (2017) [29]	Addis Ababa	Cross-sectional study	698	Active TB patients	ART status, economic status, alcohol use, perceived barrier, and psychological distress were statistically associated with non-adherence to TB medication.
Gugsa et al. (2017) [36]	SNNPR	A qualitative study	22	Active TB patients	Lack of adequate food, poor communication between healthcare providers and patients, beliefs in traditional healing, unavailability of the healthcare service in nearby, drug side-effect and pill burden, stigma, and discrimination.
Getahun et al. 2017 [32]	Addis Ababa	Mixed method design	649	Active TB patients and healthcare provider	Among lost to follow-up patients inadequate information about TB, healthcare provider–patient relationship (respect and value to patients), lack of support such as transport and nutrition support, and less commitment of healthcare provider towards the patients were reported as reasons for lost to follow-up.
Ayele et al. (2017) [15]	Amhara	Cross-sectional study	154	Latent TB-HIV patients	Lack of information about isoniazid preventive therapy (IPT), drug side effect, forgetfulness, and being away from home were found as factors for non-adherence to isoniazid preventive therapy (IPT).
Diriba et al. (2016) [26]	Oromia	Cross-sectional study	67	Active TB patients	Lack of family support, a far distance from the health facility, drug side-effects, extremely ill, feeling better, education level, and being HIV positive were the factors that led to non-adherence to TB medication.
Ayele et al. (2016) [37]	SNNPR	Prospective cohort	162	Latent TB-HIV patients	Patients experiencing a high level of HIV stigma and having opportunistic infections statistically associated with non-adherence to isoniazid preventive therapy (IPT).
Tesfahuneygn et al. 2015 [16]	Amhara	Cross-sectional study	200	Active TB patients	Forgetfulness, being away from home, drug side effects, being unable to go to the health facilities on the date of appointment, being hospitalized, TB-HIV infected, alcohol use, smoking, khat (herbal stimulant) chewing, unsatisfied with healthcare service, and long waiting time to get the health service were significantly associated.
Yasin et al. (2015) [17]	Oromia	Cross-sectional study	53	Active TB-HIV co-infected	Forgetfulness, a far distance from the health facility, and low income were the factors for non-adherence to TB medication.
Mindachew et al. (2014) [18]	Addis Ababa	A qualitative study	12	Latent TB-HIV patients	Forgetfulness, lack of patient information, knowledge about the disease and its treatment, mental health status make them reluctant to attend follow-up and clinic appointment, not disclosing their HIV status to their family members because of fear of stigma and discrimination and lack of support from family led them to non-adhere to isoniazid preventive therapy (IPT).
Kiros et al. (2014) [23]	Tigray	Cross-sectional study	278	Active TB patients	The drug side effect and knowledge about TB prevention were associated with non-adherence to TB medication.
Eticha et al. (2014) [19]	Tigray	Cross-sectional study	120	Active TB-HIV co-infected	Patients who do not have caregivers and people who do not have to remind them to take their medications more likely non-adhere to TB medications. Forgetfulness, feeling sick, and being far away from health facilities were the mention of the main reasons of the TB-HIV co-infected patients for missing medication.
Berhe et al. (2014) [34]	Addis Ababa	Cross-sectional study	381	Latent TB-HIV patients	Patients who took their medication for ≥ 5 or months were highly likely to be adherent compared to those who took it for 1-2 months. Patients’ friends’ decision to take the medication made them less likely to be non-adherent to isoniazid preventive therapy (IPT).
Nezenega et al. (2011) [39]	SNNPR	Cross-sectional study	531	Active TB patients	Employment status, area of residence, perceived time spent with a healthcare provider, perceived accessibility, perceived waiting time, perceived professional care, and overall patient satisfaction were associated with non-adherence to TB medication.
Adane et al. (2013) [20]	Amhara	Cross-sectional study	280	Active TB patients	Forgetfulness, being in the continuation phase, HIV co-infection, and persistent symptoms of tuberculosis were the factors for non-adherence to TB medication.
Tadesse et al. (2013) [30]	Amhara	A qualitative	26	Active TB	Access to health facilities, financial burdens, quality of health services, and social support were the main reason for failing to fully adhere to TB medication.
Kebede et al. (2012) [21]	SNNPR	Cross-sectional study	24	Active TB-HIV co-infected	The educational status was associated with non-adherence to TB medication. Forgetfulness and transportation cost were mentioned as reasons for non-adherence to medication.
Mindachew et al. (2011) [35]	Addis Ababa	Cross-sectional study	319	Latent TB-HIV patients	Patients who did not receive information about IPT, patients not comfortable taking IPT in front of other people, patients who did not attend regular clinic appointments, and drug side effects were associated to non-adherence to isoniazid preventive therapy (IPT).
Gebremariam et al. (2010) [31]	Addis Ababa	A qualitative study	38	Active TB-HIV co-infected and healthcare providers	Side effects, pill burden, economic constraints, lack of food, stigma with lack of disclosure, and lack of adequate communication with health professionals were barriers for adherence. While beliefs in the curability of TB, beliefs in the severity of TB in the presence of HIV infection and lack of support from families and health professionals influenced non-adherence to TB medication.
Mesfin et al. (2009) [38]	Tigray	Cross-sectional	237	Active TB patients	Lack of supervision and incapability of dealing with patients’ illness more likely to interrupt and default from treatment.
Sagbakken et al. (2008) [33]	Addis Ababa	A qualitative study	50	Active TB patients, healthcare providers and relatives	Transportation costs, poor health status due to illness or slow progression, not having social support, and not managing to restore their health and social status were factors for non-adherence.
Shargie et al. (2007) [41]	SNNPR	Prospective cohort study	404	Active TB patients	Continuation phase of treatment, a far distance from the treatment centre, and necessity to use public transport were the factors for default from TB treatment.
Michael et al. (2004) [25]	Oromia	Cross-sectional study	114	Defaulted TB patients	Far distance from the health institution, transportation cost, and being unaware about TB were the major reasons contributing to defaulting.
Tekle et al. (2002) [24]	Oromia	Case-control	1367	Active TB patients	Being in the continuation phase of treatment, lack of family support, inadequate knowledge about treatment duration and drug side effects.

SNNPR–Southern Nations, Nationalities, and Peoples’ Region, TB–Tuberculosis.
